# Aerobic capacity at age 34 predicts arterial stiffness in age 63, independent of classical and advanced lipid-related cardiovascular risk factors: a longitudinal cohort study

**DOI:** 10.1038/s41598-026-52389-8

**Published:** 2026-05-19

**Authors:** Andrea Tryfonos, Matteo Pedrelli, Paolo Parini, Eva Jansson, Ulrika Aasa, Uwe J. F. Tietge, Maria Westerståhl

**Affiliations:** 1 Division of Clinical Physiology, Department of Laboratory Medicine, Karolinska Institutet, Alfred Nobels Allé, 141 52 Huddinge, Sweden; 2 Cardio Metabolic Unit, Department of Laboratory Medicine and Department of Medicine, Karolinska Institutet, Stockholm, Sweden; 3 Medicine Unit of Endocrinology, Theme Inflammation and Ageing, Karolinska University Hospital, Stockholm, Sweden; 4 Clinical Chemistry, Karolinska University Laboratory, Karolinska University Hospital, Stockholm, Sweden; 5 Division of Clinical Chemistry, Department of Laboratory Medicine, Karolinska Institutet, Stockholm, Sweden; 6 Unit of Clinical Physiology, Karolinska University Hospital, Stockholm, Sweden

**Keywords:** Aerobic capacity, Aging, Cholesterol efflux capacity, Arterial stiffness, Risk factors, Cardiovascular diseases, Ageing

## Abstract

Atherosclerotic cardiovascular disease is the leading cause of mortality worldwide, with arterial stiffness being an important predictor of cardiovascular mortality. This study aimed to examine in the Swedish longitudinal cohort of males and females (SPAF-1958) whether aerobic capacity measured at early- (34 years) and mid-adulthood (52 years) can predict arterial stiffness assessed by pulse wave velocity later in life (63 years). Further, we determined whether this association is modified by traditional cardiovascular risk factors such as obesity, smoking, blood pressure, advanced lipoprotein profiles and high-density lipoprotein (HDL) function determined as cholesterol efflux capacity. Multiple regression analysis revealed that a higher aerobic capacity at ages 34 (B = − 0.04, P = 0.002) and 52 (B = − 0.04, P = 0.005) significantly predicted lower arterial stiffness at age 63, independent of obesity, smoking, blood pressure, HDL, and HDL-cholesterol efflux capacity. In contrast, lipoprotein profiles and HDL-mediated cholesterol efflux at age 52 were not associated with arterial stiffness at age 63 (P > 0.05). These findings suggest that maintaining aerobic capacity from early adulthood can reduce arterial stiffness and cardiovascular risk in later life, independently of traditional and contemporary cardiovascular factors. This study emphasizes the need for further research on lifestyle modifications to enhance cardiovascular health.

**Supplementary Information:**

The online version contains supplementary material available at 10.1038/s41598-026-52389-8.

## Introduction

Cardiovascular disease (CVD) and its major form, atherosclerotic cardiovascular disease, remain the leading cause of morbidity and mortality worldwide^1^. According to the World Health Organization, about 18 million people die from CVD each year, accounting for 32% of global deaths, and this number is even expected to rise to 22 million by 2030 and 32 million in 2050^2^. Arterial stiffness shares risk factors and pathomechanisms with atherosclerosis and has consequently been shown to be an important predictor of cardiovascular as well as all-cause mortality^3,4^.

While arterial stiffness increases with age^5^, it can be influenced by various factors that either promote or impair arterial health and, thus modulate cardiovascular risk^6^. For example, both physical activity and aerobic capacity are associated with a low arterial stiffness^7–11^, and therefore may have a protective effect. Regular exercise lowers traditional atherosclerotic risk factors such as blood pressure, obesity, and blood lipid levels, reduces inflammation and increases nitric oxide availability, all of which in turn improves overall vascular health^12^. Conversely, elevated levels of lipids within apolipoprotein B-containing lipoproteins, such as triglycerides or cholesterol in low-density lipoproteins (LDL)^13^, very-low density lipoproteins (VLDL) and VLDL remnants^14^, are associated with the formation of atherosclerotic plaques. Such relationships can be even better captured by performing advanced lipoprotein analyses that specifically determine the different lipid components contained within the lipoprotein subclasses directly instead of e.g. using calculation-based methods such as the Friedewald formula that derives LDL cholesterol from total cholesterol, HDL cholesterol and triglycerides^15^. In contrast, high-density lipoprotein (HDL) cholesterol has shown a positive association with arterial stiffness and cardiovascular health^16^. In fact, HDL particles play important functional roles in preventing the formation of atherosclerotic plaques such as promoting cholesterol efflux from macrophage foam cells in the vessel wall^17–19^. Cholesterol efflux is the first step in a process called reverse cholesterol transport, where cholesterol from atherosclerotic plaques is carried by HDL through the bloodstream to the liver, where it is removed from the body either directly or indirectly. By now several groups have confirmed in prospective studies that better cholesterol efflux capacity of HDL at baseline leads to fewer atherosclerotic cardiovascular events during follow-up^17–20^, positioning this parameter as a central anti-atherosclerotic function metric of HDL. Interestingly, sexually dimorphic effects have been described for the HDL cholesterol efflux capacity^18^. However, it remains unknown to which extent the above factors (aerobic capacity and/or lipid profiles and cholesterol efflux capacity) predict arterial stiffness in later life.

In the Swedish Longitudinal Physical Activity and Fitness Cohort established already in 1958 (SPAF-1958), our group has meticulously followed participants for 47 years, collecting data at ages 16, 34, 52 and 63. This comprehensive dataset includes measures of aerobic capacity and lifestyle questionnaires, supplemented over the years by additional physiological data and blood sampling/biobanking. This longitudinal dataset provides a unique opportunity to investigate the impact of aerobic capacity on the prediction of arterial stiffness in individuals aged over 60 years. The primary aim of the current work was to investigate whether aerobic capacity measured at early- (34 years) and mid-adulthood (52 years) can predict arterial stiffness later in life (63 years), independent of a number of traditional and advanced cardiovascular risk factors. The secondary aim was to comprehensively analyze detailed lipoprotein profiles and cholesterol efflux capacity in middle-aged individuals (52 years) to investigate possible sex differences and associations with arterial stiffness in later life.

## Results

The results of anthropometric measurements, blood pressure, aerobic capacity and arterial stiffness in males and females of all age groups are presented in Table 1. Females had lower blood pressure at the ages of 34 and 52, but similar values to males at the age of 63 years. There was a significant decrease with age in both absolute (L/min) and relative (mL/kg min) aerobic capacity. There was a sex difference across all ages in absolute but not in relative aerobic capacity. Arterial stiffness was measured at age 63 years and showed ~ 7% lower PWV_ao_ in males compared to females (P < 0.001), a lower proportion of males had clinically relevant arterial stiffness (PWV_ao_ ≥ 10 m/s) compared with females (24.5% vs 46.7%; P = 0.001). Using the clinically relevant cut-off for high arterial stiffness (PWVao ≥ 0 m/s), ROC analysis showed that VO₂max at 63 years of age modestly discriminated participants with elevated arterial stiffness (AUC = 0.68), with a VO₂max value of approximately 26 ml/kg/min associated with a higher likelihood of PWV_ao_ ≥ 10 m/s at 63 years (Fig. S1). Information about antihypertensive medication and lipid-lowering medications at the time of visits, as reported in participants’ medical records, as well as smoking status and participating in leisure time physical activity is also presented in Table 1. There was no sex difference at either age in none of the above variables (P > 0.05).

**Table 1:** Subjects’ characteristics at the age of 34 years, 52 years and 63 years. Data are reported as mean (SD), number of males and females for each observation (N), and sex-comparisons (P value) for each observation, with significance set to P < 0.05, unless otherwise stated. BMI, body mass index; SBP, systolic blood pressure; DPB, diastolic blood pressure; MAP, mean arterial pressure; HR, heart rate; LTPA, Leisure time physical activity; VO_2_ max, maximal volume of oxygen consumption; PWV_ao_, pulse wave velocity aortic.

	34 years					52 years					63 years				
	Males	N	Females	N	P value	Males	N	Females	N	P value	Males	N	Females	N	P value
Anthropometrics															
Height, cm	180.4 (6.9)	157	168.2 (5.9)	121	< 0.001	180.3 (7.2)	112	167.6 (6.3)	98	< 0.001	180.2 (6.7)	109	167.2 (6.2)	91	< 0.001
Weight, kg	79.7 (10.2)	157	67.2 (12.0)	121	< 0.001	86.7 (13.4)	112	72.1 (13.8)	98	< 0.001	86.3 (13.4)	109	72.4 (11.3)	91	< 0.001
BMI, m^2^/kg	24.5 (2.7)	157	23.7 (3.7)	121	0.046	26.7 (3.5)	112	25.6 (4.3)	98	0.047	26.6 (3.6)	109	25.8 (3.5)	91	0.159
Hemodynamics															
SBP, mmHg	129.5 (11.9)	157	121.4 (12.5)	121	< 0.001	131.9 (15.1)	114	126.1 (14.3)	99	0.004	134.6 (14.9)	108	131.7 (15.1)	91	0.186
DBP, mmHg	75.6 (9.0)	157	71.8 (8.9)	121	< 0.001	87.3 (10.6)	114	85.0 (10.6)	99	0.099	83.6 (9.1)	108	83.6 (10.1)	91	0.954
MAP, mmHG	93.6 (8.5)	157	88.3 (8.8)	121	< 0.001	102.1 (11.3)	114	98.7 (10.5)	99	0.021	100.6 (10.6)	108	99.6 (11.3)	91	0.522
HR, beats/min	66.7 (12.0)	157	71.2 (11.4)	121	0.002	66.5 (11.1)	111	68.0 (10.7)	94	0.351	61.0 (9.8)	104	63.3 (10.7)	82	0.128
Medications (according to patient medical records), smoking, and LTPA (data presented as N for Yes)															
Hypertension drugs, N	NA		NA		NA	17	114	20	99	0.309	47	109	31	91	0.174
Lipid-lowering drugs, N	NA		NA		NA	9	114	3	99	0.125	17	109	16	91	0.728
Smoker, N	34	157	37	121	0.089	7	114	9	99	0.399	6	109	7	91	0.530
LTPA, N	104	157	83	121	0.734	81	112	73	93	0.309	69	106	61	90	0.692
Aerobic capacity															
VO_2_ max, L/min	3.3 (0.7)	157	2.7 (0.6)	121	< 0.001	2.8 (0.7)	100	2.4 (0.6)	88	< 0.001	2.6 (0.8)	108	2.1 (0.7)	85	< 0.001
VO_2_ max, ml/kg/min	42.2 (8.7)	157	40.3 (9.6)	121	0.082	32.6 (8.1)	100	33.4(11.0)	88	0.570	27.3 (7.5)	104	26.9 (7.8)	82	0.703
Arterial stiffness															
PWV_ao_	NA		NA		NA	NA		NA		NA	9.2 (1.3)	106	9.9 (1.7)	90	< 0.001
PWV_ao_ ≥ 10 m/s, N (%)	NA		NA		NA	NA		NA		NA	26 (24.5%)		42 (46.7%)		0.001

Hierarchical multiple regression analysis was performed to examine whether aerobic capacity at age 34 years predicted arterial stiffness at 63 years. In model 1, which included VO₂ max only, aerobic capacity explained 7% of PWV_ao_ (P < 0.001). In model 2, VO₂ max was adjusted for sex, BMI, current smoking status, medications to lower lipids and blood pressure, and MAP and explained 20% in PWV_ao_ (P < 0.001). In Model 3, HDL-C and ABCA1-mediated cholesterol efflux capacity measured at the age of 52 years were added, and the model explained 36% of the variance in PWV_ao_ (P < 0.001). Across all models, VO₂ max at the age of 34 years remained a significant predictor (B =  − 0.04, P = 0.002), suggesting that higher VO₂ max at the age of 34 years is associated with lower PWV_ao_ at the age of 63 years. Similar results were found for VO_2_ max at the ages of 52 years and 63 years (Table 2). Stratified analyses by sex and antihypertensive medication use showed consistent inverse associations between VO₂ max and PWV_ao_ across subgroups (Table S1-4).

**Table 2:** Multiple linear regression using a hierarchical modeling approach with pulse wave velocity aortic (PWV_ao_) at the age of 63 years as dependent variable, and independent variables including sex and other traditional cardiovascular risk factors as reported at the age of 63 years, high-density lipoprotein (HDL) concentration and its functional capacity (cholesterol efflux capacity; CEC) as determined at the age of 52 years and aerobic capacity (VO_2_ max). Predictors were entered in three hierarchical models: Model 1 included VO₂ max only; Model 2 included Model 1 plus traditional cardiovascular risk factors; and Model 3 included Model 2 plus HDL concentration and CEC. Aerobic capacity at the ages of 34 years, 52 years and 63 years was tested in separate models following the same three-step approach. Values are presented as regression coefficients (B) with 95% confidence intervals and P values. BMI, body mass index (BMI); MAP, mean arterial pressure; HDL, high-density lipoprotein; CEC, cholesterol efflux capacity as assessed by ABCA1 in apo-depleted serum measured at the age of 52 years; VO_2_ max, maximum volume of oxygen consumption.

VO₂ max at 34 years	Model 1	Model 2	Model 3
VO₂ max (ml/kg/min)	− 0.04 (− 0.07, − 0.02), < 0.001	− 0.04 (− 0.07, − 0.01), 0.008	− 0.04 (− 0.07, − 0.02), 0.002
Female sex		0.55 (− 0.01, 1.11), 0.056	1.09 (0.49, 1.69), < 0.001
BMI (kg/m^2^)		− 0.00 (− 0.08, 0.08), 0.950	− 0.03 (− 0.11, 0.05), 0.479
MAP (mmHg)		0.03 (0.00, 0.06), 0.022	0.06 (0.04, 0.08), < 0.001
Current smoker (yes)		0.57 (− 0.44, 1.57), 0.275	0.15 (− 0.83, 1.13), 0.760
Lipid-lowering medication (yes)		− 0.05 (− 0.68, 0.58), 0.876	0.11 (− 0.60, 0.81), 0.766
Antihypertensive medication (yes)		− 0.21 (− 0.84, 0.42), 0.508	− 0.79 (− 1.35, − 0.24), 0.006
HDL cholesterol (mmol/L)			− 0.50 (− 1.13, 0.13), 0.122
Cholesterol efflux capacity (%)			− 0.11 (− 0.36, 0.14), 0.392
R^2^	0.068	0.202	0.358
N	105	105	105
VO₂ max at 52 years			
VO₂ max (ml/kg/min)	− 0.05 (− 0.08, − 0.03), < 0.001	− 0.07 (− 0.11, − 0.03), < 0.001	− 0.04 (− 0.07, − 0.01), 0.005
Female sex		1.14 (0.49, 1.80), < 0.001	0.94 (0.33, 1.54), 0.003
BMI (kg/m^2^)		− 0.09 (− 0.20, 0.01), 0.092	− 0.09 (− 0.17, 0.00), 0.046
MAP (mmHg)		0.06 (0.03, 0.09), < 0.001	0.07 (0.05, 0.10), < 0.001
Current smoker (yes)		− 0.67 (− 2.27, 0.93), 0.413	− 0.33 (− 1.45, 0.80), 0.567
Lipid-lowering medication (yes)		0.04 (− 0.64, 0.73), 0.900	0.16 (− 0.54, 0.86), 0.649
Antihypertensive medication (yes)		− 0.35 (− 1.04, 0.34), 0.318	− 0.37 (− 0.96, 0.23), 0.226
HDL cholesterol (mmol/L)			0.03 (− 0.61, 0.67), 0.935
Cholesterol efflux capacity (%)			− 0.13 (− 0.38, 0.12), 0.297
R^2^	0.093	0.389	0.335
N	114	114	114
VO₂ max at 63 years			
VO₂ max (ml/kg/min)	− 0.04 (− 0.06, − 0.02), < 0.001	− 0.04 (− 0.08, 0.00), 0.104	− 0.05 (− 0.08, − 0.02), 0.002
Female sex		0.71 (0.14, 1.29), 0.016	0.81 (0.22, 1.40), 0.007
BMI (kg/m^2^)		− 0.05 (− 0.14, 0.04), 0.220	− 0.07 (− 0.16, 0.02), 0.111
MAP (mmHg)		0.03 (0.00, 0.06), 0.030	0.06 (0.03, 0.08), < 0.001
Current smoker (yes)		0.37 (− 0.77, 1.51), 0.524	0.24 (− 0.80, 1.29), 0.646
Lipid-lowering medication (yes)		0.12 (− 0.51, 0.75), 0.711	0.28 (− 0.40, 0.95), 0.420
Antihypertensive medication (yes)		0.06 (− 0.54, 0.66), 0.852	− 0.31 (− 0.86, 0.24), 0.263
HDL cholesterol (mmol/L)			− 0.21 (− 0.80, 0.39), 0.496
Cholesterol efflux capacity (%)			− 0.15 (− 0.39, 0.10), 0.230
R^2^	0.064	0.169	0.304
N	121	121	121

**Fig. 1:**
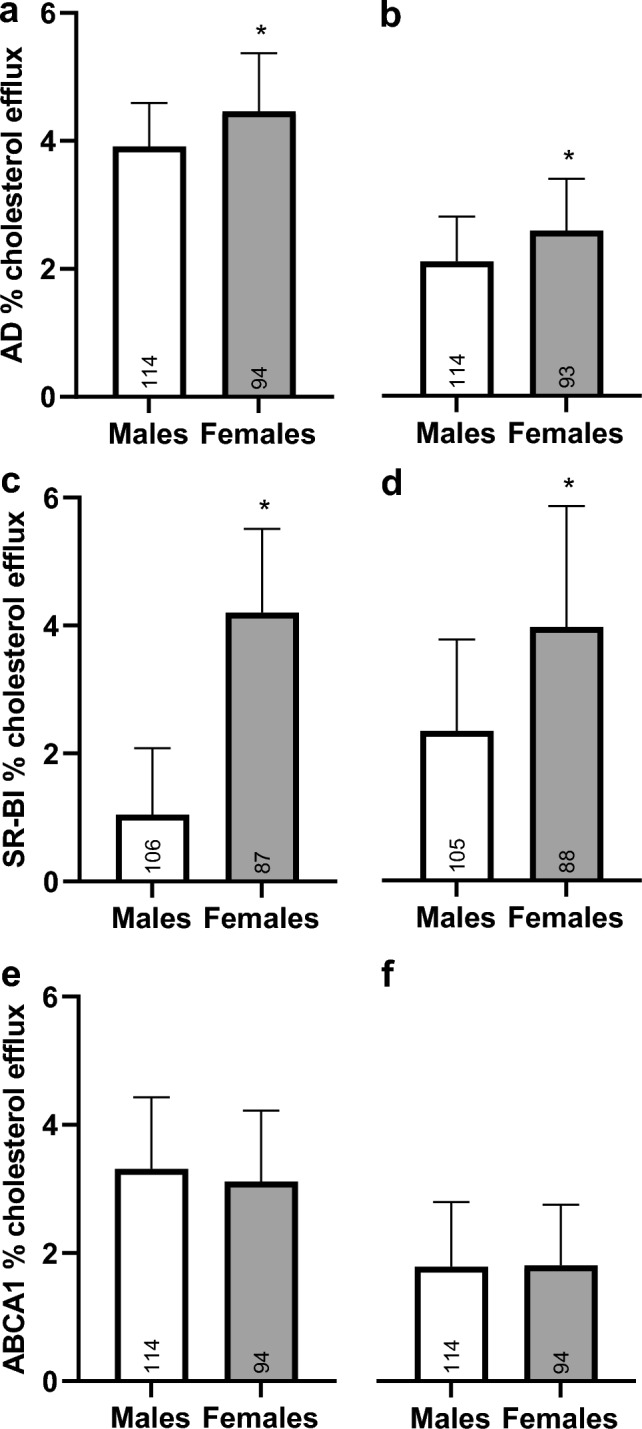
Cholesterol efflux capacity in males and females aged 52 years. AD, Aqueous diffusion; SR-BI, Scavenger receptor class B, type I; ACBA1, ATP-binding cassette transporter A1. Data are presented as mean (SD). *P < 0.05 (Mann–Whitney U test).

In the comprehensive lipoprotein analysis at age 52 years, overall HDL lipids were higher in females compared to males for all determined subspecies (P < 0.001), whereas there was no difference in LDL lipid composition between sexes (P > 0.05). In contrast, females had lower VLDL total cholesterol (P = 0.002) and unesterified cholesterol (P = 0.025), but similar VLDL triglycerides (P = 0.061), cholesteryl esters (P = 0.889) and phospholipids (P = 0.318) compared to males. When considering the circulating lipid levels, females had higher levels of cholesterol and phospholipids, but similar triglycerides compared to males (Table 3). No associations between arterial stiffness at age 63 years and cholesterol efflux capacity or lipoprotein subclasses at age 52 years were observed (P > 0.05, correlation matrix in Supplementary file). In females, both serum and apo-depleted serum showed a higher CEC via SR-BI and aqueous diffusion compared to males (Fig. 1; P < 0.001), while ABCA1-mediated cholesterol efflux was similar in both groups (Fig. 1; P > 0.05).

**Table 3:** Lipid composition of circulating lipoprotein subclasses assessed via size-exclusion chromatography at the age of 52 years. Data are presented as mean (SD), number (N) of males and females, and sex-comparisons (P value) for each observation, with significance set to P < 0.05. VLDL, Very-low-density lipoprotein; LDL, Low-density lipoprotein; HDL, High-density lipoprotein; CE, Cholesterol esters; UC, Unesterified cholesterol; TG, Triglycerides; PL, Phospholipids.

	VLDL			LDL			HDL			Total		
	Males	Females	P value	Males	Females	P value	Males	Females	P value	Males	Females	P value
CE**,** mmol/L	0.60 (0.30)	0.61 (0.30)	0.889	2.05 (0.57)	2.00 (0.61)	0.587	0.97 (0.30)	1.43 (0.55)	< 0.001	3.65 (0.88)	4.07 (1.14)	0.008
N	98	76		98	76		98	76		98	76	
UC**,** mmol/L	0.32 (0.15)	0.27 (0.11)	0.025	0.75 (0.20)	0.76 (0.19	0.802	0.30 (0.09)	0.45 (0.15)	< 0.001	1.35 (0.31)	1.45 (0.31)	0.047
N	100	78		100	78		100	78		100	78	
TC**,** mmol/L	0.92 (0.43)	0.87 (0.35)	0.002	2.80 (0.74)	2.75 (0.63)	0.612	1.26 (0.36)	1.88 (0.57)	< 0.001	4.99 (1.11)	5.50 (1.00)	0.002
N	99	81		99	81		99	81		99	81	
TG**,** mmol/L	0.81 (0.69)	0.60 (0.41)	0.061	0.37 (0.08)	0.36 (0.10)	0.559	0.22 (0.05)	0.26 (0.07)	< 0.001	1.40 (0.75)	1.20 (0.56)	0.127
N	58	49		58	49		58	49		58	49	
PL**,** mmol/L	0.48 (0.23)	0.44 (0.22)	0.318	1.22 (0.41)	1.21 (0.44)	0.839	1.25 (0.38)	1.62 (0.59)	< 0.001	2.93 (0.89)	3.25 (1.16)	0.046
N	100	84		100	84		100	84		100	84

## Discussion

The primary aim of this study was to investigate whether aerobic capacity at age 34 and 52 years can predict arterial stiffness at age 63 years, independent of traditional and more advanced cardiovascular risk factors, including the contemporary HDL function metric cholesterol efflux capacity. We found that lower aerobic capacity (VO_2_ max), but not detailed lipoprotein profiles or HDL-mediated cholesterol efflux at age 52 years predicted higher arterial stiffness at age 63 years, as measured by PWV_ao_. This association remained significant even after adjustment for a number of recognized cardiovascular risk factors. Importantly, VO_2_ max at age 34 years also predicted arterial stiffness at age 63 years.

In contrast, neither several lipid components in the main lipoprotein subclasses or specific HDL-mediated cholesterol efflux capacities (ABCA1, SR-B1, aqueous diffusion) at age 52 appeared to have substantial contributions to arterial stiffness at age 63. Finally, this study showed sex differences in the lipid contents of specific lipoprotein subclasses and in cholesterol efflux capacity. Females appeared to have higher HDL lipid levels and cholesterol efflux capacities as well as lower VLDL levels than males at age 52 years. These findings are consistent with the recognized lower CVD risk of females of that age group.

Arterial stiffness reflects a reduced elasticity of the arteries, which typically increases with age and is thought to be exacerbated by atherogenic factors. Clinically, arterial stiffness has been associated with an elevated risk of cardiovascular disease such as hypertension, heart failure, heart attack and stroke and serves as a predictor of all-cause mortality^3^, making it useful functional biomarker for assessing cardiovascular health. Physical activity and exercise are crucial for attenuating arterial stiffness as they improve endothelial function, increase nitric oxide availability and reduce inflammation^21^. All of this helps to maintain or restore the elasticity of the arteries, thereby reducing the risk of cardiovascular events and promoting overall vascular health. In the present cohort, the majority of participants (~ 65–75% across ages and both sexes) reported engaging in leisure-time physical activity; however, this was assessed using a self-reported binary question (yes/no), which provides only a crude estimate of habitual activity levels and does not capture the intensity, duration, or cumulative exposure to physical activity. Although a high level of physical activity does not necessarily translate into a high aerobic capacity (which is partly influenced by genes)^22^, people who exercise regularly, generally improve their physical fitness. Aerobic capacity has traditionally been used as a reliable metric of physical fitness and is associated with all-cause mortality and cardiovascular events in many studies^23^. Recently, Menotti et al. 2024^24^, showed that both physical activity and physical fitness predicted all-cause mortality over 60 years follow-up, with physical fitness serving as superior predictor. In addition, previous analyses from this cohort demonstrated that becoming physically active during adulthood was associated with higher aerobic capacity,^25^ further supporting the role of aerobic capacity as an integrative physiological marker reflecting cumulative physical activity exposure. There is ample cross-sectional evidence of an inverse relationship between arterial stiffness and aerobic capacity in various populations, including healthy young or middle-age adults^9,11^, the elderly^26^, generally in community-based large cohorts^10,27^, and specifically in patients with cardiovascular disease^8,28^. However, most of these studies are limited to retrospective analyses in which age is an important confounding factor.

Here, we eliminate the effect of age (all participants have the same age) and extend the results by showing that aerobic capacity in early adulthood (34 years) can predict lower arterial stiffness at age 63 years, independent of traditional cardiovascular risk factors such as obesity, smoking, arterial pressure, advanced lipid profile, and after adjustment for sex and use of medication against hypertension or dyslipidemia. Importantly, stratified analyses by sex and antihypertensive medication use showed similar effect sizes and consistent inverse associations between aerobic capacity and arterial stiffness across subgroups, supporting the robustness and independence of these findings. Aerobic capacity measured at ages 34, 52, and 63 years was inversely associated with arterial stiffness at age 63, with similar effect sizes and explained variance across separate models. Although formal statistical comparison across life stages was not performed, the consistency of these associations suggests that aerobic capacity across adulthood is relevant for later-life vascular health. This finding highlights the importance of maintaining aerobic capacity in early adulthood and midlife to achieve health benefits later in life. To further contextualize arterial stiffness at the outcome age, a secondary ROC analysis at 63 years indicated that aerobic fitness modestly discriminated clinically relevant arterial stiffness,^29^ with a VO₂ max value of approximately 26 ml/kg/min corresponding to PWV_ao_ ≥ 10 m/s. While the World Health Organization^30^ recommends at least 150 min of moderate aerobic exercise or 75 min of vigorous exercise per week for adults to improve cardiopulmonary function and overall health, lower-intensity exercise training can also significantly improve cardiopulmonary function (VO_2_ max)^31^. This is important information in favor of increasing physical activity for healthy aging and lower cardiovascular risk.

ApoB-containing lipoproteins such as VLDL and LDL substantially contribute to atherosclerotic lesion formation and represent one of the most important pathological mechanisms for the development of cardiovascular diseases. It has been well-evidenced that VLDL and LDL contribute to the production of foam cells and thus to plaque formation in the arteries^13^, while HDL has atheroprotective properties^16^. Furthermore, about a decade ago, it was suggested that more detailed information regarding the lipid composition of lipoprotein subclasses might provide more accurate information about atherosclerotic risk^32,33^, compared to the standard lipid routine. Here, we performed advanced lipoprotein profiling in ~ 200 individuals aged 52 years and associated these results with arterial stiffness at the age of 63 years. In contrast to previous studies^34–36^, our results do not support that lipoprotein subclasses are indeed related to arterial stiffness later in life. In fact, we did not observe any association between such advanced lipid parameters at the age of 52 years and arterial stiffness at the age of 63 years. Of note, our cohort consisted of relatively healthy adults at 52 years; only ~ 13% classified as obese, and 6.5% current smokers). In addition, only 68 out of 196 (~ 35%) aged 63 years, have PWV_ao_ above 10 m/s, which has been considered as a practical cut-off point for increased cardiovascular risk^29^. Analyses using this clinically relevant PWV_ao_ cut-off (≥ 10 m/s) yielded results consistent with those obtained using PWV_ao_ as a continuous variable, with no associations observed for lipid-related measures. In principle, these results regarding static HDL-C levels could point towards a relatively higher pathophysiological importance of HDL function parameters for the prediction of arterial stiffness. However, our detailed assessment of HDL-mediated cholesterol efflux did not support such a hypothesis. Since it could be questioned whether in healthy individuals any lipid parameter would associate with PWV, it is worth pointing out that for these measures no clear threshold exists between health and disease. Rather, in the case of circulating lipids a continuous, graded increase in cardiovascular risk occurs across the whole range of values, even if they are reported by clinical chemistry laboratories as ‘normal’, e.g. exemplified for LDL cholesterol and triglycerides (as biomarker of remnant cholesterol), the two major risk factors for cardiovascular disease^37,38^. Similar observations have been made for HDL function metrics including cholesterol efflux where different baseline values recorded in healthy people resulted in the differential development of cardiovascular events during follow-up^18^.

Cholesterol efflux is the process in which mainly HDL particles acquire cholesterol from peripheral tissues, most importantly macrophage foam cell within the arterial wall. It is the first and committed step of reverse cholesterol transport, an important pathway for the elimination of atherogenic cholesterol from the body^17^. HDL is particularly effective in promoting cholesterol efflux due to its interaction with key transporters such as ABCA1 and ABCG1. Reduced efficiency in cholesterol efflux can lead to cholesterol accumulation in arterial walls, conceivably contributing to arterial stiffening and increased cardiovascular risk^20^. Consequently, impaired cholesterol efflux capacity is closely associated with the development of atherosclerosis^39^ and subsequent cardiovascular events^17–19^. Cholesterol efflux mediated by SR-BI or assessed as aqueous diffusion was higher in females than in males, while there was no difference in ABCA1 receptor-mediated efflux. Although there are some observations suggesting higher cholesterol efflux capacity in females compared to males^40,41^, such data are still limited, therefore, we believe that further studies in this field are needed, specifically in relation to clinically meaningful outcomes. Importantly, we found no association between cholesterol efflux capacity and arterial stiffness.

We found sex differences in lipoprotein subclasses. Specifically, females had higher levels of all lipid species within their HDL particles, while all, except triglycerides, were lower within their VLDL. No sexually dimorphic effects were detected in the lipid composition of LDL particles. This is consistent with the cardioprotective role of estrogens in lipoprotein regulation, which appear to fluctuate over the lifespan^42^. In contrast, we found higher arterial stiffness in females than in males aged 63 years. This is not unexpected, as previous studies have indicated similar^43^ or even higher arterial stiffness^44^ in females after their 60 s, predominantly due to menopause. These observations further support our finding that lipoproteins at 52 years of age do not contribute to the level of arterial stiffness at age 63. Also, while we have no available data regarding the menopausal status at 52 years, which could in fact affect lipid metabolism, arterial stiffness at 63 years includes females after menopause. There is evidence suggesting that arterial stiffness is similar or even higher in menopausal females than in males of the same age^42^. Further studies are needed to confirm this observation.

This study has several strengths, including the long follow-up period of ~ 30 years, which provides long-term insights into the relationships between aerobic capacity, lipid profile and arterial stiffness. Objective measures were obtained for key variables such as aerobic capacity, advanced lipid profiles, functional metrics of HDL (cholesterol efflux), arterial stiffness, as well as medication and mortality from registers, which increases the reliability of the results. In addition, the inclusion of females and males contribute to the generalizability of the results to all sexes. However, the study also has certain limitations. Participants were recruited from 6 geographical areas, all of which located in Sweden, implying genotypic, phenotypic and socioeconomic similarities, which could limit the generalizability to more diverse populations. In addition, some degree of healthy participant or selection bias cannot be excluded, as individuals who remained in the study may differ from those lost to follow-up. However, drop-out analyses indicate that participants who continued in the study remained broadly representative of the original cohort in most aspects, with the exception of slightly higher physical activity levels during adolescence compared to dropouts^25^. Furthermore, it is worth noting that hard cardiovascular outcomes (i.e., cardiovascular events or diagnosis), as well as other potentially relevant factors such as pulmonary conditions, and objective measures of habitual physical activity were not included in the analyses. Finally, PWV_ao_ was assessed central arterial stiffness using an indirect, model-based and cuff-based oscillometric method, which, despite standardized procedures including appropriate cuff positioning, duplicate measurements, and a supine resting period before assessment, may still be influenced by blood pressure at the time of measurement; therefore, mean arterial pressure was included as a covariate in the primary analyses.

In conclusion, this study highlights the importance of aerobic fitness in early and middle life for cardiovascular health in later life. The results suggest that aerobic capacity (VO_2_ max) at ages 34 and 52 years is a longitudinal predictor of arterial stiffness, independent of traditional cardiovascular risk factors such as arterial pressure, smoking, BMI, as well as advanced lipid measures, and functional metrics of HDL. Unlike previous studies that have focused on either aerobic capacity, advanced lipoprotein profiles, or cholesterol efflux in relation to arterial health, to the best of our knowledge, this is the first longitudinal study that spans nearly 30 years from early to late adulthood and includes comprehensive data on both aerobic capacity, advanced lipid measures, and arterial stiffness. However, due to the observational nature of this cohort, interventional studies are needed to establish a causal relationship between aerobic capacity and arterial stiffness. Given the importance of sex differences in middle-aged adults, future studies should also consider the menopausal status of females in their 50 s. Combined, the findings of our study emphasize the importance of maintaining high aerobic fitness through regular physical activity to reduce arterial stiffness and associated cardiovascular risk. Further studies should examine these relationships in other ethnicities and more diverse populations, including also more information such as diet and sedentary behavior measures.

## Materials and methods

### Participants and ethics approval

The SPAF-1958 study has a prospective, longitudinal design including a population-based cohort of 220 teenage boys and 205 teenage girls (16 years; born in 1958) in Sweden, selected through random sampling of upper secondary school regions across six geographic areas representing different climatic conditions^45,46^, and followed up at the ages of 34, 52 and 63. The study was in accordance with the Helsinki Declaration and received ethical approval from the University of Umeå Human Research Ethics Committee (Dnr 09-082M) and the Swedish Ethical Review Authority (Dnr 2020-04338 and Dnr 2023-04163-0).

Participation was voluntary, and all participants signed an informed consent form. The study size was determined by the available cohort participants. For this report, only data from adulthood has been used (34, 52, and 63 years). At the age of 34 years, 278 participants (157 males, 121 females) were tested. At the age of 52 years, 213 of the participants (114 males, 99 females) were examined, 83% of whom participated at the age of 34 years. At the age of 63 years, 199 participants (108 males, 91 females) were examined, 80% were also participants at the age of 34 years, 75% also at the age of 52 years, and 30% (71 males, 56 females) participated at all time-points (34, 52, and 63 years). Information on medication for hypertension and hyperlipidaemia from age 52 onwards was retrieved for all participants from registers at the National Board of Health and Welfare. In the SPAF cohort, 8% died between the ages of 16 and 63. This is slightly lower than the mortality rate of 11% in the total population born in Sweden in 1958^47^. Twenty four percent of these deaths (n = 8) were due to cardiovascular disease^25^.The representativeness of these participants has already been described^25,45,48^.

### Measurements

#### Anthropometry

Height and weight were measured at the ages of 34, 52 and 63 years without shoes and in light sportswear. Body mass index (BMI, kg·m^− 2^) was calculated at all ages. Resting systolic and diastolic blood pressure as well as heart rate were also measured after supine rest at all ages^49^. Mean arterial pressure has been calculated as diastolic blood pressure + 1/3 (systolic—diastolic blood pressure).

#### Leisure-time physical activity

Leisure-time physical activity was assessed using the self-reported question: “Do you engage in any physical activity during your free time (including, for example, walking or gardening)?”, with response options yes/no, as previously described^25^.

#### Aerobic capacity

A submaximal exercise test on a cycle ergometer (Monark 828E, Monark Exercise AB, Sweden, Lode Corival model 906900) was performed at all ages to determine maximal aerobic capacity (VO_2_ max). Absolute aerobic capacity (L min^− 1^) was estimated using an Åstrand nomogram and adjusted for age^45^. The relative aerobic capacity was calculated (ml·kg^− 1^ min^− 1^).

#### Lipoprotein analysis and cholesterol efflux capacity

Blood samples were taken following overnight fasting at the age of 52 years and stored in the freezer at − 80 °C or further analysis. Lipoproteins and cholesterol efflux experiments were performed on serum samples as previously described^50^. Briefly, samples were thawed at most twice on ice and apoB-depleted serum was prepared by polyethylene glycol precipitation. Lipoproteins were separated from 2.5 μl of individual serum samples by size exclusion chromatography, using a Superose 6 PC 3.2/300 column (GE Healthcare Bio-Sciences AB, Uppsala, Sweden). Triglycerides (TG), total cholesterol (TC), unesterified cholesterol (UC), and phospholipid (PL) concentrations were calculated after integration of the individual chromatograms, generated by the enzymatic-colorimetric reaction with the respective kits: Cholesterol CHOD-PAP, TG GPO-PAP (Roche Diagnostics, Mannheim, Germany), and free cholesterol E, PL C (FujiFilm Wako Diagnostics, Mountain View, CA). The amount of esterified cholesterol was calculated by subtracting the UC from the TC.

Whole and apoB-depleted serum samples were tested as cholesterol acceptors in different cell models in order to measure their cholesterol efflux capacity (CEC)^50^. J774A.1 cells cultured under basal conditions were used to assess aqueous diffusion. ABCA1-mediated cholesterol efflux was the difference between the cholesterol efflux of J774A.1 incubated with 8-(4-Chlorophenylthio) adenosine 3′,5′-cyclic monophosphate sodium salt (cpt-cAMP; 0.3 mmol/L) and aqueous diffusion. The cholesterol efflux via scavenger receptor class B type I (SR-BI) was the difference between the cholesterol effluxes measured from Fu5AH cells cultured under basal conditions and Fu5AH cells incubated with block lipid transporter 1 (BLT-1;10 µmol/L). Briefly, cells were plated in 24-well plates in medium containing 10% fetal bovine serum (FBS). The monolayers were washed with PBS and incubated for 24 h in medium containing 1% FBS, [1,2-3H(N)]-cholesterol (2 mCi/mL) and a Sterol *O*-acyltransferase (SOAT) inhibitor. Cells were then incubated for 18 h with medium plus 0.2% bovine serum albumin (BSA) and SOAT inhibitor (2 µg/mL), with compounds added as needed. Several wells of cells were harvested with NaOH 1 M and counted by liquid scintillation to provide the initial values (time 0) for total [1,2-3H(N)]-cholesterol content. The remaining cell monolayers were incubated for 4 h with 1% (v/v) serum or 1.4% apoB-depleted (apoBd) serum (v/v) in the medium. The cell media were filtered, and radioactivity was determined by liquid scintillation counting. Cholesterol efflux was calculated as: (cpm in medium at 4 h/cpm at time 0) × 100.

#### Arterial stiffness

Measurements of central (aortic) pulse wave velocity (PWV_ao_) were performed at 63 years of age using a non-invasive technique (Arteriograph, TensioMed Ltd., Budapest, Hungary), as previously described^51^. Participants were rested in supine position for at least 10 min, and then a single upper arm cuff was placed to record brachial pressure waveforms for the estimation of PWV_ao_. The device first measures the brachial blood pressure and then inflates the cuff to suprasystolic pressure to occlude the brachial artery. During this period, the cuff collects pure pressure signals and detects direct and reflected systolic wave peaks. The time difference between the early and late systolic peaks is equal to the time of the aortic pulse wave traveling down to the aortic bifurcation and back towards the heart. The aortic root-bifurcation transit time can be calculated, and by estimating the straight distance between the suprasternal notch and pubic bone (an acceptable estimate of the aortic length), the PWV_ao_ was calculated by the TensioMed Arteriograph software. A cut-off value of 10 m/s was applied to define clinically relevant arterial stiffness, in accordance with previous literature.^29^.

#### Statistics

Statistical analysis was performed using SPSS version 29.0 (IBM Corp, Armonk, NY). Differences in continuous variables between males and females at each age were assessed by independent Student’s t-test for normally distributed data and Mann–Whitney test for non-normally distributed data. The Chi-square test was used to test differences between sexes for categorical data. Receiver operating characteristic (ROC) curve analysis was used to evaluate the discriminatory ability of aerobic capacity (VO₂ max) for high arterial stiffness (PWV_ao_ ≥ 10 m/s), accounting for the inverse association between fitness and arterial stiffness, and to identify a VO₂ max value associated with higher likelihood of PWV_ao_ ≥ 10 m/s at the age of 63 years. This ROC analysis was included as a sensitivity analysis based on a clinically relevant arterial stiffness cut-off. A correlation matrix was used to assess the associations between the dependent variable (arterial stiffness at 63 years) and the independent variables (lipoprotein subclasses, cholesterol efflux measures at 52 years), to select the most suitable independent variables for multiple regression. None of the lipoprotein subclasses or cholesterol efflux measures were significantly associated with arterial stiffness (Supplementary material). However, given the relevance of HDL cholesterol and ABCA1-mediated cholesterol efflux capacity in atheroprotection^39^, both variables have been included in the multiple linear regression. Hierarchical multiple regression was performed to assess whether aerobic capacity (VO_2_ max) at ages of 34 years, 52 years and 63 years (separately) predicted arterial stiffness (PWV_ao_) at age of 63 years, independent of traditional cardiovascular risk factors such as sex, obesity (BMI), smoking status, mean arterial blood pressure, anti-hypertensive and lipid-lowering medications (according to medical records), HDL, and cholesterol efflux capacity. Independent variables were entered hierarchically in 3 models: Model 1 included aerobic capacity (VO_2_ max); Model 2 included Model 1 plus sex, BMI, anti-hypertensive and lipid-lowering medications, smoking status, mean arterial pressure; Model 3 induced Model 2 plus HDL-C, and cholesterol efflux capacity. Models 1 and 2 are presented for descriptive purposes, with inference based on the fully adjusted model. To assess whether sex modified the association between aerobic capacity and arterial stiffness, a sex × VO₂ max interaction term was tested in the fully adjusted model, sex- and antihypertensive medication use-stratified analyses were additionally performed (Supplementary Material). Aerobic capacity (VO_2_ max) at the age of 34 years, 52 years and 63 years (separate analysis for each one). Model fit was assessed using R^2^. Analyses were conducted using complete-case data; no imputation was performed, and the number of participants included in each analysis is reported in the corresponding tables and figures. Statistical significance was set at P < 0.05.

## Supplementary Information

Supplementary Information 1.

Supplementary Information 2.

## Data Availability

Data are available from the corresponding author upon reasonable request.
