# Supplementary material for: Aerobic capacity at age 34 predicts arterial stiffness in age 63, independent of classical and advanced lipid-related cardiovascular risk factors: a longitudinal cohort study
Source: Sci Rep. 2026 May 19;16:15467. doi: 10.1038/s41598-026-52389-8 (PMC13186957; doi:10.1038/s41598-026-52389-8)
Supplement: Supplementary file 1 — Supplementary Information 1. [file 41598_2026_52389_MOESM1_ESM.docx]

STROBE Statement—checklist of items that should be included in reports of observational studies

|  | Item No. | Recommendation | Page  No. | Relevant text from manuscript |
| --- | --- | --- | --- | --- |
| **Title and abstract** | 1 | (*a*) Indicate the study’s design with a commonly used term in the title or the abstract | 1 | Aerobic capacity at age 34 predicts arterial stiffness in age 63, independent of classical and advanced lipid-related cardiovascular risk factors: a longitudinal cohort study |
|  |  | (*b*) Provide in the abstract an informative and balanced summary of what was done and what was found | 1 | “This study aimed to examine in the Swedish longitudinal cohort… whether aerobic capacity measured at early- (34 years) and mid-adulthood (52 years) can predict arterial stiffness assessed by pulse wave velocity later in life (63 years)…Multiple regression analysis revealed that higher aerobic capacity… predicted lower arterial stiffness…” |
| Introduction | | | |  |
| Background/rationale | 2 | Explain the scientific background and rationale for the investigation being reported | 3 | “Arterial stiffness shares risk factors and pathomechanisms with atherosclerosis and has consequently been shown to be an important predictor of cardiovascular as well as all-cause mortality.”  “However, it remains unknown to which extent the above factors (aerobic capacity and/or lipid profiles and cholesterol efflux capacity) predict arterial stiffness in later life.” |
| Objectives | 3 | State specific objectives, including any prespecified hypotheses | 3 | “The primary aim… The secondary aim…” |
| Methods | | | |  |
| Study design | 4 | Present key elements of study design early in the paper | 9 | “The SPAF-1958 study has a prospective, longitudinal design…” |
| Setting | 5 | Describe the setting, locations, and relevant dates, including periods of recruitment, exposure, follow-up, and data collection | 9 | “Population-based cohort… selected through random sampling… followed up at the ages of 34, 52 and 63.” |
| Participants | 6 | (*a*) *Cohort study*—Give the eligibility criteria, and the sources and methods of selection of participants. Describe methods of follow-up  *Case-control study*—Give the eligibility criteria, and the sources and methods of case ascertainment and control selection. Give the rationale for the choice of cases and controls  *Cross-sectional study*—Give the eligibility criteria, and the sources and methods of selection of participants | 9 | “At the age of 34 years, 278 participants… at the age of 52 years, 213 participants… at the age of 63 years, 199 participants…” |
|  |  | (*b*) *Cohort study*—For matched studies, give matching criteria and number of exposed and unexposed  *Case-control study*—For matched studies, give matching criteria and the number of controls per case | 9 | “Participants were followed up at the ages of 34, 52 and 63.” |
| Variables | 7 | Clearly define all outcomes, exposures, predictors, potential confounders, and effect modifiers. Give diagnostic criteria, if applicable | 9 | “PWVao was calculated… Aerobic capacity (VO₂ max)… HDL cholesterol and cholesterol efflux capacity…” |
| Data sources/ measurement | 8* | For each variable of interest, give sources of data and details of methods of assessment (measurement). Describe comparability of assessment methods if there is more than one group | *9-10* | *Aerobic capacity:*  *“A submaximal exercise test on a cycle ergometer… was performed…”*  *PWV:*  *“Measurements of central pulse wave velocity were performed using Arteriograph…”*  *Cholesterol efflux:*  *“Whole and apoB-depleted serum samples were tested as cholesterol acceptors…”* |
| Bias | 9 | Describe any efforts to address potential sources of bias | 8 | “Healthy participant and selection bias may be present, as individuals who remained in the study may be healthier…” |
| Study size | 10 | Explain how the study size was arrived at | 9 | “The study size was determined by the available cohort participants.” |

Continued on next page

| Quantitative variables | 11 | Explain how quantitative variables were handled in the analyses. If applicable, describe which groupings were chosen and why | 10 | “Hierarchical multiple regression was performed…” |
| --- | --- | --- | --- | --- |
| Statistical methods | 12 | (*a*) Describe all statistical methods, including those used to control for confounding | 10 | “Hierarchical multiple regression… Model 1 included aerobic capacity… Model 2 included confounders…” |
|  |  | (*b*) Describe any methods used to examine subgroups and interactions | 10 | “Sex × VO₂ max interaction term was tested… sex-stratified analyses were additionally performed.” |
|  |  | (*c*) Explain how missing data were addressed | 10 | “Analyses were conducted using complete-case data; no imputation was performed…” |
|  |  | (*d*) *Cohort study*—If applicable, explain how loss to follow-up was addressed  *Case-control study*—If applicable, explain how matching of cases and controls was addressed  *Cross-sectional study*—If applicable, describe analytical methods taking account of sampling strategy | 10 | “Analyses were conducted using complete-case data; no imputation was performed…” |
|  |  | (*e*) Describe any sensitivity analyses | 10 | “ROC analysis was included as a sensitivity analysis based on a clinically relevant arterial stiffness cut-off.” |
| Results | | | | |
| Participants | 13* | (a) Report numbers of individuals at each stage of study—eg numbers potentially eligible, examined for eligibility, confirmed eligible, included in the study, completing follow-up, and analysed | 4, Tables, Figures | “At the age of 63 years, 199 participants were examined…” |
|  |  | (b) Give reasons for non-participation at each stage | NA |  |
|  |  | (c) Consider use of a flow diagram | NA |  |
| Descriptive data | 14* | (a) Give characteristics of study participants (eg demographic, clinical, social) and information on exposures and potential confounders | 4, Tables, Figures | “The results of anthropometric measurements, blood pressure, aerobic capacity and arterial stiffness… are presented in Table 1.” |
|  |  | (b) Indicate number of participants with missing data for each variable of interest | 4, Tables, Figures |  |
|  |  | (c) *Cohort study*—Summarise follow-up time (eg, average and total amount) | 8 | ..followed up at the ages of 34, 52 and 63 |
| Outcome data | 15* | *Cohort study*—Report numbers of outcome events or summary measures over time | 4, Tables, Figures |  |
|  |  | *Case-control study—*Report numbers in each exposure category, or summary measures of exposure | *NA* |  |
|  |  | *Cross-sectional study—*Report numbers of outcome events or summary measures | *NA* |  |
| Main results | 16 | (*a*) Give unadjusted estimates and, if applicable, confounder-adjusted estimates and their precision (eg, 95% confidence interval). Make clear which confounders were adjusted for and why they were included | 4 | “VO₂ max at age 34 years remained a significant predictor (B=−0.04, P=0.002)…” |
|  |  | (*b*) Report category boundaries when continuous variables were categorized | NA |  |
|  |  | (*c*) If relevant, consider translating estimates of relative risk into absolute risk for a meaningful time period | NA |  |

Continued on next page

| Other analyses | 17 | Report other analyses done—eg analyses of subgroups and interactions, and sensitivity analyses | 4, 10 | “ROC analysis showed that VO₂ max at 63 years modestly discriminated participants with elevated arterial stiffness…” |
| --- | --- | --- | --- | --- |
| Discussion, 10 | | | | |
| Key results | 18 | Summarise key results with reference to study objectives | 4 | “We found that lower aerobic capacity… predicted higher arterial stiffness…” |
| Limitations | 19 | Discuss limitations of the study, taking into account sources of potential bias or imprecision. Discuss both direction and magnitude of any potential bias | 7 | “Healthy participant and selection bias may be present…” |
| Interpretation | 20 | Give a cautious overall interpretation of results considering objectives, limitations, multiplicity of analyses, results from similar studies, and other relevant evidence | 4-7 |  |
| Generalisability | 21 | Discuss the generalisability (external validity) of the study results | 7 | “Participants were recruited from Sweden… which could limit generalizability…” |
| Other information | |  | | |
| Funding | 22 | Give the source of funding and the role of the funders for the present study and, if applicable, for the original study on which the present article is based | 10 | “This study has been funded by the Swedish Research Council…” |

*Give information separately for cases and controls in case-control studies and, if applicable, for exposed and unexposed groups in cohort and cross-sectional studies.

**Note:** An Explanation and Elaboration article discusses each checklist item and gives methodological background and published examples of transparent reporting. The STROBE checklist is best used in conjunction with this article (freely available on the Web sites of PLoS Medicine at http://www.plosmedicine.org/, Annals of Internal Medicine at http://www.annals.org/, and Epidemiology at http://www.epidem.com/). Information on the STROBE Initiative is available at www.strobe-statement.org.
