# Supplementary material for: Aerobic capacity at age 34 predicts arterial stiffness in age 63, independent of classical and advanced lipid-related cardiovascular risk factors: a longitudinal cohort study
Source: Sci Rep. 2026 May 19;16:15467. doi: 10.1038/s41598-026-52389-8 (PMC13186957; doi:10.1038/s41598-026-52389-8)
Supplement: Supplementary file 2 — Supplementary Information 2. [file 41598_2026_52389_MOESM2_ESM.docx]

**Supplementary material**

**Figure S1**. Receiver operating characteristic (ROC) curve illustrating the ability of aerobic fitness, assessed by maximal oxygen consumption (VO₂max), to discriminate between participants with high arterial stiffness, defined as aortic pulse wave velocity (PWVao ≥10 m/s), and low arterial stiffness (PWVao <10 m/s).

**Figure S2.** Spearman correlation matrix depicting associations between lipoprotein subclasses and cholesterol efflux capacity measured at the age of 52 years and arterial stiffness measured at the age of 63 years.

**Table S1.** Hierarchical multiple linear regression analysis examining the association between aerobic capacity and pulse wave velocity aortic (PWVao) at the age of 63 years in men.

**Table S2.** Hierarchical multiple linear regression analysis examining the association between aerobic capacity and pulse wave velocity aortic (PWVao) at the age of 63 years in women.

**Table S3.** Hierarchical multiple linear regression analysis examining the association between aerobic capacity and pulse wave velocity aortic (PWVao) at the age of 63 years in participants using antihypertensive medication.

**Table S4.** Hierarchical multiple linear regression analysis examining the association between aerobic capacity and pulse wave velocity aortic (PWVao) at the age of 63 years in participants not using antihypertensive medication.

**Table S5**. Lipid composition of circulating lipoprotein subclasses assessed via size-exclusion chromatography at the age of 52 years stratified by arterial stiffness status at the age of 63 years (PWVao <10 m/s vs ≥10 m/s).

**Figure S1.** Receiver operating characteristic (ROC) curve illustrating the ability of aerobic fitness, assessed by maximal oxygen consumption (VO₂max), to discriminate between participants with high arterial stiffness, defined as aortic pulse wave velocity (PWVao ≥10 m/s), and low arterial stiffness (PWVao <10 m/s). VO₂max values were inverted prior to analysis to account for the inverse relationship between fitness and arterial stiffness.The area under the curve (AUC) was 0.68.


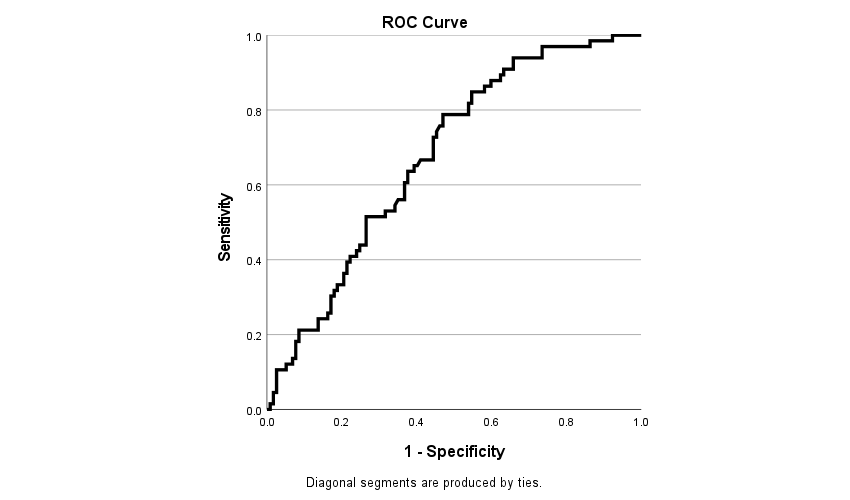


AUC= 0.667

**Figure S2.** Spearman correlation matrix depicting associations between lipoprotein subclasses and cholesterol efflux capacity measured at the age of 52 years and arterial stiffness measured at the age of 63 years. Spearman correlations were assessed and none were found statistically significant (P>0.05).

ABCA1; ATP-binding cassette transporter ABCA1, AD; Aqueous diffusion, CE; Cholesterol Esters, HDL; High-Density Lipoprotein, LDL; Low-Density Lipoprotein, PL; Phospholipids, PWVao; pulse wave velocity aortic, SR-BI; Scavenger receptor class B, type I, TG; Triglycerides, UC; Unesterified Cholesterol, VLDL; Very-Low-Density Lipoprotein

**Table S1:** Hierarchical multiple linear regression analysis examining the association between aerobic capacity and pulse wave velocity aortic (PWVao) at the age of 63 years in men. A hierarchical modeling approach was used with pulse wave velocity aortic (PWVao) at the age of 63 years as the dependent variable, and independent variables including traditional cardiovascular risk factors as reported at the age of 63 years, high-density lipoprotein (HDL) concentration and its functional capacity (cholesterol efflux capacity; CEC) as determined at the age of 52 years, and aerobic capacity (VO₂ max) added in three steps. Predictors were entered in three hierarchical models: Model 1 included VO₂ max only; Model 2 included Model 1 plus traditional cardiovascular risk factors (BMI, mean arterial pressure, smoking status, lipid-lowering medication, and antihypertensive medication); and Model 3 included Model 2 plus HDL concentration and CEC. Aerobic capacity at the ages of 34, 52, and 63 years was tested in separate models following the same three-step approach. Values are presented as regression coefficients (B) with 95% confidence intervals and P values. Abbreviations: BMI, body mass index; MAP, mean arterial pressure; HDL, high-density lipoprotein; CEC, cholesterol efflux capacity as assessed by ABCA1 in apo-depleted serum measured at the age of 52 years; VO₂ max, maximum volume of oxygen consumption.

| **VO₂ max at 34 years** | **Model 1** | **Model 2** | **Model 3** |
| --- | --- | --- | --- |
| VO₂ max (ml/kg/min) | -0.02 (-0.05, 0.02), 0.421 | -0.02 (-0.06, 0.01), 0.238 | -0.03 (-0.06, 0.01), 0.190 |
| BMI (kg/m²) |  | -0.02 (-0.12, 0.08), 0.707 | -0.04 (-0.15, 0.07), 0.480 |
| MAP (mmHg) |  | 0.06 (0.03, 0.09), <0.001 | 0.06 (0.03, 0.09), <0.001 |
| Current smoker (yes) |  | 0.21 (-1.34, 1.77), 0.789 | 0.30 (-1.25, 1.84), 0.710 |
| Lipid-lowering medication (yes) |  | 0.34 (-0.55, 1.23), 0.458 | 0.22 (-0.68, 1.11), 0.638 |
| Antihypertensive medication (yes) |  | -0.71 (-1.41, 0.00), 0.057 | -0.74 (-1.44, -0.03), 0.046 |
| HDL cholesterol |  |  | -0.76 (-1.68, 0.17), 0.114 |
| Cholesterol efflux capacity |  |  | -0.01 (-0.32, 0.30), 0.950 |
| R² | 0.011 | 0.239 | 0.275 |
| N | 62 | 62 | 62 |
| **VO₂ max at 52 years** |  |  |  |
| VO₂ max (ml/kg/min) | -0.034 (-0.077, 0.009), 0.115 | -0.059 (-0.104, -0.014), 0.014 | -0.056 (-0.103, -0.009), 0.021 |
| BMI (kg/m²) |  | -0.089 (-0.197, 0.019), 0.112 | -0.096 (-0.206, 0.014), 0.092 |
| MAP (mmHg) |  | 0.065 (0.034, 0.096), <0.001 | 0.066 (0.035, 0.097), <0.001 |
| Current smoker |  | -0.161 (-1.708, 1.386), 0.839 | -0.075 (-1.629, 1.479), 0.925 |
| Lipid lowering medication |  | 0.017 (-0.890, 0.924), 0.972 | -0.076 (-0.996, 0.844), 0.872 |
| Antihypertensive medication |  | -0.819 (-1.550, -0.088), 0.032 | -0.858 (-1.590, -0.126), 0.026 |
| HDL cholesterol |  |  | -0.541 (-1.427, 0.345), 0.236 |
| CEC |  |  | -0.080 (-0.380, 0.220), 0.605 |
| R² | 0.040 | 0.285 | 0.310 |
| N | 64 | 64 | 64 |
| **VO₂ max at 63 years** |  |  |  |
| VO₂ max (ml/kg/min) | -0.042 (-0.075, -0.009), 0.015 | -0.047 (-0.082, -0.012), 0.014 | -0.043 (-0.080, -0.006), 0.027 |
| BMI (kg/m²) |  | -0.078 (-0.192, 0.036), 0.180 | -0.085 (-0.201, 0.031), 0.151 |
| MAP (mmHg) |  | 0.051 (0.020, 0.082), 0.002 | 0.053 (0.022, 0.084), 0.001 |
| Current smoker |  | -0.060 (-1.638, 1.518), 0.941 | 0.041 (-1.534, 1.616), 0.960 |
| Lipid lowering medication |  | 0.093 (-0.781, 0.967), 0.836 | 0.009 (-0.869, 0.887), 0.985 |
| Antihypertensive medication |  | -0.417 (-1.097, 0.263), 0.234 | -0.452 (-1.129, 0.225), 0.196 |
| HDL cholesterol |  |  | -0.648 (-1.502, 0.206), 0.142 |
| CEC |  |  | -0.067 (-0.369, 0.235), 0.666 |
| R² | 0.158 | 0.207 | 0.269 |
| N | 69 | 69 | 69 |

**Table 2. Hierarchical multiple linear regression analysis examining the association between aerobic capacity and pulse wave velocity aortic (PWVao) at the age of 63 years in women.** A hierarchical modeling approach was used with pulse wave velocity aortic (PWVao) at the age of 63 years as the dependent variable, and independent variables including traditional cardiovascular risk factors as reported at the age of 63 years, high-density lipoprotein (HDL) concentration and its functional capacity (cholesterol efflux capacity; CEC) as determined at the age of 52 years, and aerobic capacity (VO₂ max) added in three steps.Predictors were entered in three hierarchical models: Model 1 included VO₂ max only; Model 2 included Model 1 plus traditional cardiovascular risk factors (BMI, mean arterial pressure, smoking status, lipid-lowering medication, and antihypertensive medication); and Model 3 included Model 2 plus HDL concentration and CEC. Aerobic capacity at the ages of 34, 52, and 63 years was tested in separate models following the same three-step approach. Values are presented as regression coefficients (B) with 95% confidence intervals and P values. Abbreviations: BMI, body mass index; MAP, mean arterial pressure; HDL, high-density lipoprotein; CEC, cholesterol efflux capacity as assessed by ABCA1 in apo-depleted serum measured at the age of 52 years; VO₂ max, maximum volume of oxygen consumption.

| **VO₂ max at 34 years** | **Model 1** | **Model 2** | **Model 3** |
| --- | --- | --- | --- |
| VO₂ max (ml/kg/min) | -0.07 (-0.11, -0.03), 0.002 | -0.07 (-0.11, -0.03), 0.002 | -0.06 (-0.11, -0.02), 0.006 |
| BMI (kg/m²) |  | 0.01 (-0.13, 0.14), 0.946 | 0.03 (-0.12, 0.17), 0.727 |
| MAP (mmHg) |  | 0.06 (0.01, 0.10), 0.013 | 0.06 (0.01, 0.10), 0.013 |
| Current smoker (yes) |  | -0.25 (-1.66, 1.16), 0.730 | -0.10 (-1.54, 1.33), 0.889 |
| Lipid-lowering medication (yes) |  | -0.15 (-1.38, 1.08), 0.814 | -0.13 (-1.36, 1.10), 0.839 |
| Antihypertensive medication (yes) |  | -0.77 (-1.66, 0.12), 0.100 | -0.83 (-1.76, 0.10), 0.090 |
| HDL cholesterol |  |  | -0.27 (-1.17, 0.63), 0.556 |
| Cholesterol efflux capacity |  |  | -0.28 (-0.73, 0.18), 0.243 |
| R² | 0.219 | 0.397 | 0.426 |
| N | 43 | 43 | 43 |
| **VO₂ max at 52 years** |  |  |  |
| VO₂ max (ml/kg/min) | -0.03 (-0.08, 0.01), 0.062 | -0.06 (-0.11, -0.01), 0.014 | -0.06 (-0.10, -0.01), 0.021 |
| BMI (kg/m²) |  | -0.089 (-0.197, 0.019), 0.112 | -0.096 (-0.206, 0.014), 0.092 |
| MAP (mmHg) |  | 0.065 (0.034, 0.096), <0.001 | 0.066 (0.035, 0.097), <0.001 |
| Current smoker |  | -0.161 (-1.708, 1.386), 0.839 | -0.075 (-1.629, 1.479), 0.925 |
| Lipid lowering medication |  | 0.017 (-0.890, 0.924), 0.972 | -0.076 (-0.996, 0.844), 0.872 |
| Antihypertensive medication |  | -0.819 (-1.550, -0.088), 0.032 | -0.858 (-1.590, -0.126), 0.026 |
| HDL cholesterol |  |  | -0.541 (-1.427, 0.345), 0.236 |
| CEC |  |  | -0.080 (-0.380, 0.220), 0.605 |
| R² | 0.040 | 0.285 | 0.310 |
| N | 64 | 64 | 64 |
| **VO₂ max at 63 years** |  |  |  |
| Predictor | Model 1 | Model 2 | Model 3 |
| VO₂ max (ml/kg/min) | -0.069 (-0.122, -0.016), 0.012 | -0.068 (-0.125, -0.011), 0.023 | -0.066 (-0.123, -0.009), 0.029 |
| BMI (kg/m²) |  | -0.094 (-0.249, 0.061), 0.238 | -0.084 (-0.241, 0.073), 0.299 |
| MAP (mmHg) |  | 0.061 (0.020, 0.102), 0.007 | 0.065 (0.022, 0.108), 0.005 |
| Current smoker |  | 0.300 (-1.302, 1.902), 0.715 | 0.488 (-1.157, 2.133), 0.564 |
| Lipid lowering medication |  | 0.504 (-0.598, 1.606), 0.375 | 0.637 (-0.492, 1.766), 0.275 |
| Antihypertensive medication |  | -0.015 (-0.950, 0.920), 0.976 | -0.040 (-0.993, 0.913), 0.935 |
| HDL cholesterol |  |  | 0.090 (-0.787, 0.967), 0.842 |
| CEC |  |  | -0.272 (-0.701, 0.157), 0.220 |
| R² | 0.120 | 0.275 | 0.301 |
| N | 52 | 52 | 52 |

**Table S3. Hierarchical multiple linear regression analysis examining the association between aerobic capacity and pulse wave velocity aortic (PWVao) at the age of 63 years in participants using antihypertensive medication.** A hierarchical modeling approach was used with pulse wave velocity aortic (PWVao) at the age of 63 years as the dependent variable, and independent variables including traditional cardiovascular risk factors as reported at the age of 63 years, high-density lipoprotein (HDL) concentration and its functional capacity (cholesterol efflux capacity; CEC) as determined at the age of 52 years, and aerobic capacity (VO₂ max) added in three steps. Predictors were entered in three hierarchical models: Model 1 included VO₂ max only; Model 2 included Model 1 plus traditional cardiovascular risk factors (sex, BMI, mean arterial pressure, smoking status, and lipid-lowering medication); and Model 3 included Model 2 plus HDL concentration and CEC. Aerobic capacity at the ages of 34, 52, and 63 years was tested in separate models following the same three-step approach. Values are presented as regression coefficients (B) with 95% confidence intervals and P values. Abbreviations: BMI, body mass index; MAP, mean arterial pressure; HDL, high-density lipoprotein; CEC, cholesterol efflux capacity as assessed by ABCA1 in apo-depleted serum measured at the age of 52 years; VO₂ max, maximum volume of oxygen consumption.

| **VO₂ max at 34 years** | **Model 1** | **Model 2** | **Model 3** |
| --- | --- | --- | --- |
| VO₂ max (ml/kg/min) | -0.067, p=0.013 | -0.060, p=0.034 | -0.061, p=0.040 |
| Sex |  | 0.747, p=0.066 | 0.962, p=0.056 |
| BMI (kg/m²) |  | 0.035, p=0.487 | 0.023, p=0.686 |
| MAP (mmHg) |  | 0.041, p=0.034 | 0.043, p=0.031 |
| Smoking |  | 0.021, p=0.979 | -0.067, p=0.937 |
| Lipids |  | 0.408, p=0.327 | 0.363, p=0.396 |
| HDL cholesterol |  |  | -0.461, p=0.429 |
| CEC |  |  | -0.007, p=0.971 |
| R² | 0.142 | 0.327 | 0.339 |
| N | 43 | 43 | 43 |
| **VO₂ max at 52 years** |  |  |  |
| VO₂ max (ml/kg/min) | -0.024, p=0.465 | -0.104, p=0.003 | -0.109, p=0.004 |
| Sex |  | 1.895, p<0.001 | 1.644, p=0.012 |
| BMI |  | -0.179, p=0.013 | -0.176, p=0.026 |
| MAP |  | 0.073, p=0.002 | 0.070, p=0.004 |
| Smoking |  | -1.150, p=0.253 | -1.136, p=0.276 |
| Lipids |  | 0.500, p=0.293 | 0.557, p=0.267 |
| HDL |  |  | 0.463, p=0.532 |
| CEC |  |  | 0.040, p=0.856 |
| R² | 0.014 | 0.450 | 0.458 |
| N | 40 | 40 | 40 |
| **VO₂ max at 63 years** |  |  |  |
| VO₂ max | -0.037, p=0.087 | -0.042, p=0.099 | -0.038, p=0.154 |
| Sex |  | 1.000, p=0.030 | 1.164, p=0.044 |
| BMI |  | -0.038, p=0.565 | -0.034, p=0.646 |
| MAP |  | 0.041, p=0.068 | 0.044, p=0.062 |
| Smoking |  | 0.157, p=0.864 | 0.151, p=0.873 |
| Lipids |  | 0.412, p=0.380 | 0.429, p=0.372 |
| HDL |  |  | -0.274, p=0.671 |
| CEC |  |  | -0.129, p=0.587 |
| R² | 0.064 | 0.247 | 0.257 |
| N | 47 | 47 | 47 |

**Table S4. Hierarchical multiple linear regression analysis examining the association between aerobic capacity and pulse wave velocity aortic (PWVao) at the age of 63 years in participants not using antihypertensive medication.** A hierarchical modeling approach was used with pulse wave velocity aortic (PWVao) at the age of 63 years as the dependent variable, and independent variables including traditional cardiovascular risk factors as reported at the age of 63 years, high-density lipoprotein (HDL) concentration and its functional capacity (cholesterol efflux capacity; CEC) as determined at the age of 52 years, and aerobic capacity (VO₂ max) added in three steps. Predictors were entered in three hierarchical models: Model 1 included VO₂ max only; Model 2 included Model 1 plus traditional cardiovascular risk factors (sex, BMI, mean arterial pressure, smoking status, and lipid-lowering medication); and Model 3 included Model 2 plus HDL concentration and CEC. Aerobic capacity at the ages of 34, 52, and 63 years was tested in separate models following the same three-step approach. Values are presented as regression coefficients (B) with 95% confidence intervals and P values. Abbreviations: BMI, body mass index; MAP, mean arterial pressure; HDL, high-density lipoprotein; CEC, cholesterol efflux capacity as assessed by ABCA1 in apo-depleted serum measured at the age of 52 years; VO₂ max, maximum volume of oxygen consumption.

| **VO₂ max at 34 years** | **Model 1** | **Model 2** | **Model 3** |
| --- | --- | --- | --- |
| VO₂ max | -0.048, p=0.012 | -0.044, p=0.010 | -0.041, p=0.018 |
| Sex |  | 0.720, p=0.033 | 0.943, p=0.024 |
| BMI |  | -0.123, p=0.049 | -0.114, p=0.069 |
| MAP |  | 0.083, p<0.001 | 0.084, p<0.001 |
| Smoking |  | -0.149, p=0.817 | 0.062, p=0.925 |
| Lipids |  | -0.593, p=0.429 | -0.630, p=0.402 |
| HDL |  |  | -0.339, p=0.410 |
| CEC |  |  | -0.182, p=0.270 |
| R² | 0.100 | 0.403 | 0.427 |
| N | 62 | 62 | 62 |
| **VO₂ max at 52 years** |  |  |  |
| VO₂ max | -0.047, p=0.018 | -0.035, p=0.052 | -0.033, p=0.062 |
| Sex |  | 0.632, p=0.044 | 0.666, p=0.073 |
| BMI |  | -0.095, p=0.106 | -0.093, p=0.113 |
| MAP |  | 0.075, p<0.001 | 0.079, p<0.001 |
| Smoking |  | -0.212, p=0.752 | -0.077, p=0.911 |
| Lipids |  | -0.173, p=0.761 | -0.102, p=0.859 |
| HDL |  |  | -0.035, p=0.923 |
| CEC |  |  | -0.208, p=0.175 |
| R² | 0.076 | 0.337 | 0.357 |
| N | 74 | 74 | 74 |
| **VO₂ max at 63 years** |  |  |  |
| VO₂ max | -0.074, p<0.001 | -0.062, p=0.005 | -0.060, p=0.007 |
| Sex |  | 0.459, p=0.143 | 0.526, p=0.161 |
| BMI |  | -0.121, p=0.050 | -0.120, p=0.052 |
| MAP |  | 0.065, p<0.001 | 0.070, p<0.001 |
| Smoking |  | 0.148, p=0.826 | 0.283, p=0.681 |
| Lipids |  | -0.047, p=0.934 | 0.011, p=0.984 |
| HDL |  |  | -0.079, p=0.825 |
| CEC |  |  | -0.206, p=0.175 |
| R² | 0.152 | 0.350 | 0.370 |
| N | 74 | 74 | 74 |

**Table S5**. Lipid composition of circulating lipoprotein subclasses assessed via size-exclusion chromatography at the age of 52 years stratified by arterial stiffness status at the age of 63 years (PWVao <10 m/s vs ≥10 m/s).Participants were stratified according to pulse wave velocity aortic (PWVao) measured at the age of 63 years using a clinically relevant cut-off value of 10 m/s to define lower and higher arterial stiffness groups. Lipid composition of circulating lipoprotein subclasses was assessed via size-exclusion chromatography at the age of 52 years.

Data are presented as mean (SD), number (N) of participants in each PWVao category, and between-group comparisons (P value) for each observation, with statistical significance set at P < 0.05. Abbreviations: PWVao, pulse wave velocity aortic; VLDL, very-low-density lipoprotein; LDL, low-density lipoprotein; HDL, high-density lipoprotein; CE, cholesterol esters; UC, unesterified cholesterol; TG, triglycerides; PL, phospholipids.

|  | | VLDL |  | |  | LDL |  | |  | | | HDL |  |  | | | Total | |  |  |
| --- | --- | --- | --- | --- | --- | --- | --- | --- | --- | --- | --- | --- | --- | --- | --- | --- | --- | --- | --- | --- |
|  | PWV ≥10 | | PWV <10 | P value | | PWV ≥10 | PWV <10 | | P value | | | PWV ≥10 | PWV <10 | P value | | | PWV ≥10 | | PWV <10 | P value |
| CE, mmol/L | 0.62 (0.35) | | 0.59 (0.31) | 0.684 | | 2.04 (0.64) | | 1.95 (0.61) | | 0.420 | 1.31 (0.56) | | 1.15 (0.48) | | 0.101 | 4.01 (1.13) | | 3.71 (1.06) | | 0.146 |
| N | 45 | | 78 |  | | 45 | | 78 | |  | 45 | | 78 | |  | 45 | | 78 | |  |
| UC, mmol/L | 0.29 (0.15) | | 0.29 (0.14) | 0.865 | | 0.75 (0.20) | | 0.72 (0.17) | | 0.280 | 0.38 (0.17) | | 0.35 (0.13) | | 0.299 | 1.38 (0.39) | | 1.34 (0.27) | | 0.503 |
| N | 46 | | 75 |  | | 46 | | 75 | |  | 46 | | 75 | |  | 46 | | 75 | |  |
| TC, mmol/L | 0.90 (0.48) | | 0.88 (0.39) | 0.727 | | 2.79 (0.80) | | 2.65 (0.62) | | 0.275 | 1.72 (0.69) | | 1.51 (0.50) | | 0.050 | 5.41 (1.34) | | 5.04 (0.92) | | 0.070 |
| N | 48 | | 80 |  | | 48 | | 80 | |  | 48 | | 80 | |  | 48 | | 80 | |  |
| TG, mmol/L | 0.59 (0.40) | | 0.74 (0.74) | 0.371 | | 0.37 (0.10) | | 0.36 (0.09) | | 0.781 | 0.25 (0.07) | | 0.24 (0.06) | | 0.756 | 1.21 (0.49) | | 1.34 (0.82) | | 0.462 |
| N | 25 | | 46 |  | | 25 | | 46 | |  | 25 | | 46 | |  | 25 | | 46 | |  |
| PL, mmol/L | 0.48 (0.27) | | 0.46 (0.22) | 0.563 | | 1.26 (0.47) | | 1.19 (0.41) | | 0.373 | 1.55 (0.63) | | 1.42 (0.49) | | 0.193 | 3.29 (1.18) | | 3.03 (1.03) | | 0.183 |
| N | 48 | | 82 |  | | 48 | | 82 | |  | 48 | | 82 | |  | 48 | | 82 | |  |
